# Evolving moldy murderers: *Aspergillus* section *Fumigati* as a model for studying the repeated evolution of fungal pathogenicity

**DOI:** 10.1371/journal.ppat.1008315

**Published:** 2020-02-27

**Authors:** Antonis Rokas, Matthew E. Mead, Jacob L. Steenwyk, Nicholas H. Oberlies, Gustavo H. Goldman

**Affiliations:** 1 Department of Biological Sciences, Vanderbilt University, Nashville, Tennessee, United States of America; 2 Department of Chemistry and Biochemistry, University of North Carolina at Greensboro, Greensboro, North Carolina, United States of America; 3 Faculdade de Ciencias Farmacêuticas de Ribeirão Preto, Universidade de São Paulo, São Paulo, Brazil; McGill University, CANADA

“*Biology is more like history than it is like physics*. *You have to know the past to understand the present*.”–Carl Sagan (1980)

## Introduction

Species in the genus *Aspergillus* are saprophytic filamentous fungi that are most commonly found in soil and litter environments of subtropical and warm temperate latitudes [1]. Inhalation of asexual spores produced by *Aspergillus fumigatus* and a few other species in the genus cause a group of diseases collectively referred to as aspergillosis [2]. The most severe form of aspergillosis is invasive aspergillosis, which primarily affects individuals with compromised immune systems or preexisting lung conditions [3]. Since drugs targeting invasive aspergillosis are not always effective due to our lack of understanding of how they function inside the human host [4] and the evolution of drug resistance [5, 6], infected individuals suffer high morbidity and mortality [7]. Collectively, *Aspergillus* fungi affect millions of patients and cause hundreds of thousands of life-threatening infections every year [8, 9].

Not all pathogenic *Aspergillus* species exhibit the same infection rates [10, 11]. Approximately 70% of all *Aspergillus* infections are caused by *A*. *fumigatus*, whereas the remaining 30% of infections stem from other species in the genus [12]. Some of these other pathogenic species–for example, *Aspergillus flavus*, *Aspergillus niger*, and *Aspergillus terreus*–are distantly related to *A*. *fumigatus* [12]; each of these three species belong to different *Aspergillus* sections (note “section” is a taxonomic rank in-between the genus and species ranks) and show extensive genomic divergence [13]. However, there are also pathogenic *Aspergillus* species, such as *Aspergillus lentulus* and *Aspergillus udagawae*, that belong to the same section as *A*. *fumigatus* (section *Fumigati*) and are much more closely related [14–16]. In contrast, the vast majority of *Aspergillus* species, including many close relatives of the pathogens mentioned above, are either not pathogenic or very rarely cause disease [16, 17]. Interestingly, *A*. *fumigatus* and a few other species in the genus can also cause opportunistic infections, mainly in other mammals and birds, and occasionally in other vertebrates and invertebrates [18].

Even though a great deal is known about some aspects of *A*. *fumigatus* pathogenicity [2, 19, 20], we have only recently begun to examine why pathogenicity varies so dramatically across the entire genus and the traits and genetic elements that contributed to this variation. Addressing this question requires that we consider the fact that pathogenic *Aspergillus* species are not dependent on their hosts for survival and their pathogenic effects are entirely accidental or opportunistic. Thus, understanding the evolution of pathogenicity in the genus requires that we understand how variation in the traits that enable *Aspergillus* species to survive in their natural soil and litter environments has rendered a few of these species capable to establish infections inside human hosts.

In this pearl article, we focus on section *Fumigati*, a lineage of ~60 species that includes *A*. *fumigatus* and its close relatives [16, 21], to discuss the latest advances in our understanding of the evolution of pathogenicity in *Aspergillus*. More broadly, given that the ability to cause human disease has repeatedly evolved across the fungal tree of life, and that the vast majority of human fungal pathogens have non-pathogenic close relatives [22], understanding the evolution of *Aspergillus* pathogenicity can serve as a model for studying fungal pathogenicity in general.

## Pathogenicity in *Aspergillus* section *Fumigati* fungi has evolved multiple times independently

**Fig 1:**
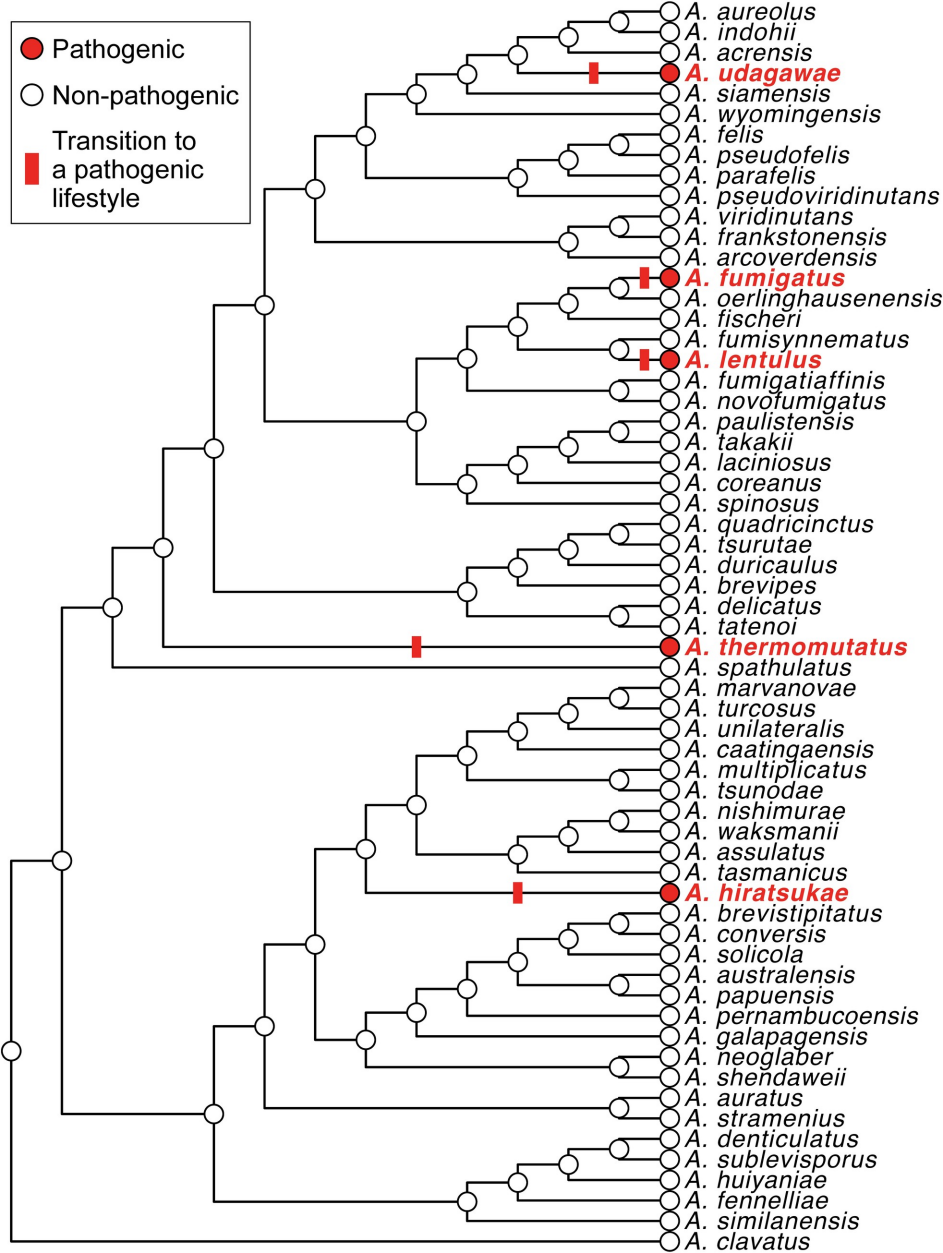
Reconstruction of the ability to cause human disease on the *Aspergillus* section *Fumigati* phylogeny suggests that pathogenicity evolved multiple times independently in the lineage. For the trait reconstruction inference, Biosafety Level (BSL) 2 organisms were considered pathogenic and BSL1 organisms or organisms that so far lack BSL labelling were considered non-pathogenic; these transitions to a pathogenic lifestyle (i.e., from BSL1 to BSL2) are labelled by red bars on the figure. Note that clinical isolates from humans or other mammals from a few additional species in the section have been identified [10, 28]; this handful includes relatively newly described species that some authors consider to have pathogenic potential (e.g., *Aspergillus novofumigatus* [29]) as well as organisms thought to be on the non-pathogenic end of the spectrum (e.g., *A*. *fischeri* [30, 31]). The phylogeny of the section was redrawn from Hubka *et al*. [16].

Only a handful of species in section *Fumigati* are considered pathogenic [10, 16, 21], and the distribution of these pathogens on the section’s phylogeny [16] suggests that the ability to cause human disease has evolved at least 5 times independently (**Fig 1**). For example, whereas *A*. *fumigatus* infects >300,000 humans per year [9], its close evolutionary relative *A*. *fischeri*, whose protein sequences exhibit, on average, 95% similarity to their *A*. *fumigatus* orthologs, has only rarely been reported to cause human disease and is not considered clinically relevant [23, 24]. Interestingly, evolutionary reconstruction shows that *A*. *fumigatus* pathogenicity likely evolved after the species diverged from either *A*. *fischeri* or from its even closer non-pathogenic relative *A*. *oerlinghausenensis*, suggesting that the last common ancestor of these three species was non-pathogenic (**Fig 1**). The same is true for most other pathogens in the section. For example, the pathogenic *A*. *udagawae* accounts for a few thousand infections per year [25–27], but its close relatives (e.g., *Aspergillus aureolus*, *Aspergillus acrensis*, and *Aspergillus wyomingensis*) are not considered clinically relevant (**Fig 1**), suggesting that the pathogenicity of *A*. *udagawae* evolved independently and that the common ancestor of *A*. *udagawae* and its close relatives was non-pathogenic.

## The observed spectrum of pathogenicity cannot be explained by ecology or ascertainment bias and is likely to have a genetic component

Several ecological attributes, such as the global ubiquity of their small and readily airborne asexual spores, are thought to contribute to the pathogenicity of *Aspergillus* molds in general [11], and to the pathogenicity of *A*. *fumigatus* in particular [19, 32]. Although these ecological attributes are undoubtedly important for infecting and causing disease in a human host, the observed spectrum of pathogenicity among section *Fumigati* species cannot be solely explained by the known differences in species’ ecologies [1, 33–35]. For example, previous studies have shown that *A*. *fischeri*, one of the closest non-pathogenic relatives of *A*. *fumigatus*, can be frequently isolated from a variety of locales, including soils, fruits, and hospitals [33–35]. Case in point, approximately 2% of the fungi isolated from the Beijing Hospital environment were *A*. *fischeri* [34], but only a handful of infections caused by this fungus have ever been reported [10, 23, 24].

Another ecological attribute known to be associated with fungal pathogenicity is thermal tolerance [36]. However, all species in section *Fumigati* that have been tested can grow, dependent on the growth medium used, at 37°C [14]. These data suggest that pathogenicity in *Aspergillus* section *Fumigati* is not simply due to species’ abilities to grow at the human body temperature. Nevertheless, species in the section do show substantial differences in how well they can grow at 37°C [14], but these differences are likely to have a genetic basis (see below). It would be highly interesting for future studies to examine growth curves of closely related pathogenic and non-pathogenic species in specific stressful and human infection-relevant conditions (e.g., at 37°C, with limited nutrient availability, low levels of oxygen and high levels of oxidative stress).

Another potential explanation for some of the observed differences in the spectrum of pathogenicity among species is ascertainment bias. In the context of *Aspergillus* pathogenicity, ascertainment bias is a term that describes systematic deviations from the true incidence of disease caused by a given species. These systematic deviations stem from the methods used to taxonomically identify (ascertain) clinical isolates and estimate how often they cause disease, i.e., from our failure to measure the true numbers of infections caused by so-called cryptic species, namely organisms that are morphologically similar to major pathogens, such as *A*. *fumigatus*, but genetically distinct from them [10]. The true burden some of these cryptic species, including species currently thought to not be clinically relevant, place on human health is unknown and may be in several cases underestimated [22]. However, numerous molecular typing studies of clinical isolates from diverse countries routinely identify the known pathogens in section *Fumigati* (Fig 1), but not the non-pathogens [25–27], indicating that the observed variation in pathogenicity is not solely an artifact of species misdiagnosis. These data suggest that the differences in pathogenicity observed across *Aspergillus* section *Fumigati* have, at least partially, a genetic basis.

Support for the role of genetic differences in contributing to the observed spectrum of pathogenicity is provided by the numerous traits, and their underlying genes and pathways, that are required for pathogenicity in *A*. *fumigatus* [2, 19, 20, 37] and have been found to exhibit substantial genetic and phenotypic diversity among section *Fumigati* species. These traits include thermotolerance, the ability to respond to multiple environmental stresses, including antifungal drugs, and the capacity to biosynthesize a range of structurally diverse secondary metabolites [14, 30, 38, 39].

## Two models for the evolution of pathogenicity in *Aspergillus* molds

**Fig 2:**
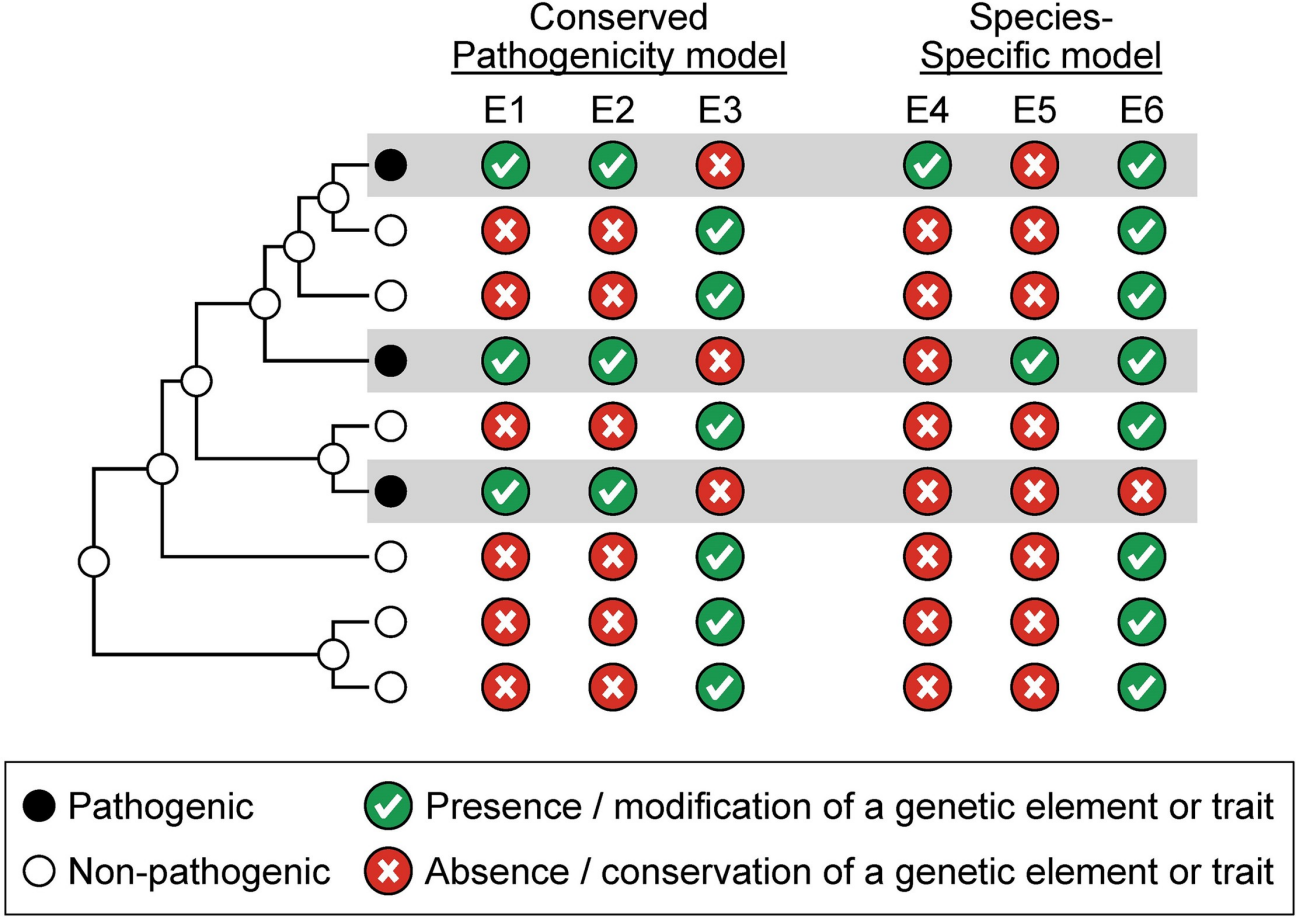
Graphic illustration of the “conserved pathogenicity” and the “species-specific” pathogenicity models.

One useful approach for gaining insights into the genetic foundations of the multiple, independent origins of pathogenicity in *Aspergillus* section *Fumigati* is the development of conceptual models that describe the differences that we would expect to observe in genomic comparisons involving pathogenic and non-pathogenic species. We propose two alternative, although not necessarily mutually exclusive, models, which we have named the “conserved pathogenicity” model and the “species-specific pathogenicity” model (**Fig 2**). The conserved pathogenicity model posits that *A*. *fumigatus* and other pathogenic species in section *Fumigati* share common pathogenicity traits and genetic elements (or shared differences in genetic elements) that are absent in non-pathogens (e.g., traits / elements E1 and E2 in **Fig 2**) or vice versa (e.g., trait / element E3 in **Fig 2**). In contrast, the species-specific model posits that each pathogen contains a unique suite of traits and genetic elements (or unique differences in genetic elements) that distinguish it from its non-pathogenic relatives; these traits / elements could be ones that are uniquely present in a given pathogen but absent in related pathogens and non-pathogens (e.g., traits / elements E4 and E5 in **Fig 2**) or vice versa (e.g., trait / element E6 in **Fig 2**). Note that these shared or species-specific genetic elements (or differences in genetic elements) are not limited to differences in gene content but to any type of genetic variation that alters pathogenicity trait values. These variants can range from, for example, differences in a single or in a handful of nucleotides within otherwise conserved protein-coding or non-coding (regulatory) regions to larger-scale differences concerning the presence of entire genetic pathways and networks.

Discerning which model explains the repeated evolution of pathogenicity is key for developing research strategies to understand the underlying molecular mechanisms in the genus and, more broadly, in filamentous fungi and beyond. For example, the conserved pathogenicity model would predict that pathogenicity stems from the action of conserved genetic elements, suggesting that known genetic determinants of virulence in *A*. *fumigatus* [40] would be great candidates for involvement in virulence in other pathogenic *Aspergillus* species. It is the adoption of the conserved pathogenicity model that underlies recent examinations of the degree to which genetic elements known to contribute to *A*. *fumigatus* pathogenicity are conserved in other species [29, 30, 41]. In contrast, the species-specific model would predict the opposite, namely that the genetic determinants of virulence are unique to each pathogen, suggesting that extrapolations of knowledge on virulence mechanisms from one pathogenic species to another would be futile. Genomic comparisons between the major pathogen *A*. *fumigatus* and its close non-pathogenic relative *A*. *fischeri* [30, 31] as well as broad comparisons of select species across the genus [29, 41] have begun to shed light on the validity of, and provide support for, both of these models.

## Support for the conserved pathogenicity model

Consistent with one of the predictions of the conserved pathogenicity model, examinations of dozens of *A*. *fumigatus* genes known to be associated with virulence [40] have shown that most of these genetic determinants of virulence are highly conserved in closely related species [29, 30]. For example, a recent genomic comparison of the major pathogen *A*. *fumigatus* with its close, non-pathogenic relative *A*. *fischeri* showed that 48 of 49 known genetic determinants of *A*. *fumigatus* virulence (e.g., CrzA, the C_2_H_2_-type zinc finger transcription factor involved in calcium ion homeostasis, or LaeA, a methyltransferase and master regulator of secondary metabolism) were highly conserved in *A*. *fischeri* [30]. However, these results also suggested that the differences in virulence among organisms spanning the pathogenicity spectrum may not be primarily due to differences in gene content, which virtually all genomic comparisons of fungal pathogens and non-pathogens in the genus [29–31, 41] and beyond [42–44] have heavily focused on. How these conserved genetic determinants of virulence function in other pathogenic, as well as in non-pathogenic, species is an interesting future direction of inquiry.

## Support for the species-specific pathogenicity model

Comparisons of gene content between closely related *Aspergillus* species have also identified numerous genes that appear to be species-specific. For example, a broad scale comparison of *A*. *fumigatus* strains Af293 and A1163 against *A*. *fischeri* and *A*. *clavatus* found that approximately 8.5% of genes were unique to *A*. *fumigatus* and absent from the other two species [31]. These *A*. *fumigatus*-specific genes tend to reside near the ends of chromosomes (i.e., are subtelomeric) and have functions associated with metabolism (e.g., secondary metabolism, transport, and detoxification), raising the hypothesis that some of them may aid *A*. *fumigatus* survival inside the human host [31]. For example, more than two thirds of *A*. *fumigatus* biosynthetic gene clusters are absent from the closely related non-pathogen *A*. *fischeri* [30, 38]; however some of these biosynthetic gene clusters are found in other species in section *Fumigati*, suggesting that they were lost in *A*. *fischeri* rather than originated within *A*. *fumigatus*. Several other clusters and their secondary metabolites appear to have uniquely evolved in *A*. *fumigatus*, or their loss in species closely related to *A*. *fumigatus* was so widespread that they are now uniquely present in *A*. *fumigatus*.

One recent, striking example in support of the species-specific model was the discovery of the gene hypoxia-responsive morphology factor A or *hrmA* (Afu5g14900) [45]. While homologs of *hmrA* are present in other distantly related fungi, this subtelomeric gene is polymorphic within *A*. *fumigatus* and is absent from the genomes of all sequenced *Aspergillus* section *Fumigati* species. Investigation of *hrmA* function shows that it likely regulates a cluster of genes, which also appear to be absent from the genomes of other *Aspergillus* section *Fumigati* species, that collectively contribute to the generation of a morphotype that facilitates adaptation to very low oxygen conditions encountered by the fungus inside human lungs [45].

## Concluding remarks

This pearl has focused on *Aspergillus* section *Fumigati*, outlining two general models for the repeated evolution of pathogenicity in the section and how they could aid in the design of experiments aimed at elucidating the underlying molecular changes responsible. The same approach could also be employed for developing strategies for the burgeoning problem of drug resistance (e.g., to what extent are mechanisms of drug resistance conserved across pathogenic species?). But the utility of studying the evolution of *Aspergillus* section *Fumigati* pathogenicity goes beyond, as Carl Sagan so aptly put it, understanding “the present”. By posing questions such as “are species that are currently considered non-pathogenic but contain conserved genetic determinants of virulence likely to emerge as new pathogens?”, we believe that an evolutionary approach–by identifying the presence of constellations of genes and traits associated with pathogenicity in non-pathogens–also holds promise for predicting the emergence of new pathogens *in the future*.
